# The role of cut-off values for creatinine, blood urea nitrogen, and uric acid in prognostic assessment of chronic heart failure: a retrospective cohort study

**DOI:** 10.1186/s12872-025-04675-y

**Published:** 2025-03-22

**Authors:** Zheng Xu, Yuebing Yue, Manfei Xu, Liyan Qian, Liping Dou

**Affiliations:** 1 The Second Affiliated Hospital of Zhejiang Chinese Medical University, Hangzhou, China; 2 The Second Clinical Medical College of Zhejiang Chinese Medical University, Binwen Road 548, Binjiang District, Hangzhou, 310053 China

**Keywords:** Chronic heart failure, Long-term prognosis, Cardiorenal syndrome, Renal function, Primary healthcare

## Abstract

**Background:**

Chronic heart failure (CHF) significantly harms patients and society, causing high mortality and reduced quality of life, straining healthcare systems; early identification and intervention are crucial for improving long-term prognosis.

**Methods:**

This retrospective cohort study involved 297 CHF patients. After collecting data on demographics, lab results, echocardiography, and comorbidities, ROC analysis was used to determine optimal cut-off values, followed by survival analysis and multivariate Cox regression to identify poor prognosis risk factors.

**Results:**

ROC analysis set optimal cut-offs for Scr, BUN, and UA at 101.5 µmol/L, 8.61 mmol/L, and 462 µmol/L, with AUCs of 0.602 (Scr, UA) and 0.674 (BUN). Kaplan-Meier analysis showed significant curve separation, while Cox regression identified risk factors for poor prognosis: Scr ≥ 101.5 µmol/L (HR = 2.209, 95% CI 1.372–3.557, *P* = 0.001), BUN ≥ 8.61 mmol/L (HR = 3.709, 95% CI 2.270–6.061, *P* < 0.001), UA ≥ 462 µmol/L (HR = 2.625, 95% CI 1.631–4.228, *P* < 0.001), male sex(HR = 1.764, 95% CI 1.067–2.915, *P* = 0.027), hyperlipidemia (HR = 0.567, 95% CI 0.351–0.916, *P* = 0.02), and re-hospitalization(HR = 0.480, 95% CI 0.280–0.826, *P* = 0.008). Subgroup analysis indicates that male gender is a significant risk factor for females (OR:2.424, *P* < 0.001); and age also posed a risk (OR:1.026, *P* = 0.036). NYHA class IV had an OR of 0.42 compared to class III (*P* < 0.001), and class III had an OR of 0.307 compared to class II (*P* = 0.016). Patients without CHD had a 1.905-fold increased risk of poor prognosis (*P* = 0.033).

**Conclusion:**

This study highlights key characteristics, assessment parameters, and risk factors for CHF patients, emphasizing the importance of Scr, BUN, and UA cut-off levels in management and guiding future research.

**Supplementary Information:**

The online version contains supplementary material available at 10.1186/s12872-025-04675-y.

## Introduction

Chronic heart failure (CHF) is a prevalent and serious cardiovascular condition, with its incidence and mortality rates increasing globally [1]. The disease not only substantially impairs patients’ quality of life but also imposes a significant economic burden on society. Consequently, the early identification of high-risk individuals and the provision of effective prognostic assessments are of paramount importance. Biomarkers such as the serum N-terminal pro-brain natriuretic peptide (NT-pro BNP), the New York Heart Association (NYHA) classification, and the left ventricular ejection fraction (LVEF) are integral components of the current evaluation framework for CHF patients [2, 3]. Additionally, clinical experience shows that liver and kidney function indicators [4, 5], along with comorbid conditions [6], significantly affect the long-term prognosis of CHF.

In recent years, the rapid advancement in biomarker research and novel laboratory indices has facilitated a shift towards more precise prognostic evaluations for CHF. Research indicates that emerging biomarkers like miR-122 [7–9], extracellular vesicles [10–12], CA-125 [13, 14], lactate [15], hyaluronic acid (HA) [16] and lactate glucose index (LGI) [17] possess predictive value in assessing the prognosis of CHF. Despite the promising findings regarding the clinical utility of these new biomarkers, additional research is necessary to validate their efficacy and reliability. Furthermore, due to limitations in infrastructure and technical capabilities, primary healthcare facilities face challenges in implementing comprehensive testing for these advanced biomarkers. Thus, optimizing, integrating, and exploring the predictive potential of widely utilized laboratory indicators becomes crucial for enhancing the long-term prognostic assessment of CHF patients.

Our previous research showed that patients with heart failure complicated by cardiorenal syndrome frequently exhibit a poor prognosis [18–20]. To strengthen scientific rigor and integrity, we collected the clinical data from complete blood count, liver and renal function, cardiac function, comorbidities, and readmissions to conduct this retrospective analysis of CHF patients’ prognosis, focusing on specific biochemical markers–serum creatinine (Scr), blood urea nitrogen (BUN), and uric acid (UA) levels. Although previous studies have demonstrated that these biochemical markers are closely linked to the prognosis of patients with heart failure, offering valuable insights for clinical decision-making, in-depth analysis of their association with prognosis and potential in therapeutic strategy merits further exploring.

This study used a retrospective cohort design to analyze clinical data from 297 CHF patients, allowing us to utilize existing data while minimizing research duration and expenses. Additionally, Kaplan-Meier survival analysis and Cox proportional hazards regression were employed to comprehensively assess the influence of individual risk factors on patient outcomes, identifying the critical determinants of long-term prognosis. By examining the relationship between these biomarkers and CHF, this study aims to elucidate the potential utility of these biomarkers in patient care management.

## Methods

### Study design and data collection

This study recruited 297 patients diagnosed with CHF who were hospitalized in the Cardiology Department of our institution from January 1st, 2020, to December 31st, 2023. Patients’ ages ranges from 39 to 91 years old and included both males and females. The primary underlying conditions were either coronary atherosclerotic heart disease or idiopathic dilated cardiomyopathy. Medical records of the patients included in the study were systematically reviewed and collected via the hospital’s electronic health record system. The following data were documented: (1) The patient demographics, including gender, age, and contact information; (2) The clinical presentation, including admission date, duration of illness, cardiac function classification, and left ventricular ejection fraction; (3) The laboratory test results consist of blood routine examination, liver and renal function, serum lipid levels, NT-proBNP levels and UA levels; (4) The relevant medical history, including the prevalence of coronary artery disease, diabetes mellitus, hypertension, and dyslipidemia.

### Study population

The diagnoses of coronary atherosclerotic heart disease and idiopathic dilated cardiomyopathy align with the clinical diagnosis of chronic heart failure. Additionally, one of the following criteria must be met: (1) LVEF < 50%; (2) NT-proBNP ≥ 900 pg/mL.

The following conditions were grounds for exclusion from the study: (1) acute myocardial infarction or heart failure; (2) cardiogenic shock, life-threatening arrhythmias, advanced atrioventricular block, or uncontrolled hypertension; (3) severe primary diseases affecting the liver, kidneys, hematological system, primary thyroid disorders, or malignancies; (4) psychiatric disorders that prevent cooperation or willingness to participate; (5) an insufficient follow-up period of less than 30 days.

The work described has been carried out in accordance with The Code of Ethics of the World Medical Association. Informed consent to participate was obtained from all the participants in the study during the follow-up procedure.

### Endpoints

The patient received treatment in accordance with the 2014 Chinese Guidelines for the Diagnosis and Treatment of Chronic Heart Failure [21] and received symptomatic management for related complications during hospitalization. Post-discharge follow-up was conducted via telephone. The primary endpoint was mortality, The primary endpoint was mortality, and the date of each patient’s death was recorded. No further interventions were administered during hospitalization or in the post-discharge follow-up.

### Statistical analysis

Analyses were performed using SPSS 27.0, Prism 9.0, and R Studio (version 4.2.1). Normally distributed data was presented as mean ± standard deviation (mean ± SD), whereas non-normally distributed data are shown as median with interquartile range [M (Q1-Q3)]. Categorical data are reported as counts and percentages [N (%)]. Distribution patterns were assessed using frequency statistics. Inter-group differences were evaluated using chi-square tests and non-parametric tests. Spearman correlation analysis was employed to examine the influencing factors. Receiver operating characteristic (ROC) curve analysis was utilized to determine the cut-off values of risk factors. Kaplan-Meier survival analysis was applied to depict the survival curves of different groups, and the log-rank test was used to assess the differences. Multivariable Cox regression analysis was conducted to develop a risk regression model for each risk factor, and univariate binary logistic regression analysis was performed to conduct subgroup analyses for patients in different groups. Statistical significance thresholds were defined as follows: *P* < 0.05 indicates significance, *P* < 0.01 indicates high significance, and *P* > 0.05 indicates non-significance.

## Results

### Baseline characteristics

A total of 297 patients were enrolled in this study, comprising 153 (51.5%) males and 144 (48.5%) females. Participants’ ages ranged from 24 to 98 years old, with a median age of 77 years old (IQR 66–82). Disease duration ranged from 1 month to 50 years, with a median of 7 years (IQR 2-16.5). Among the participants, 238 (80.1%) were diagnosed with coronary heart disease (CHD), 109 (36.7%) with diabetes mellitus (DM), 213 (71.7%) with hypertension (HT), and 182 (61.3%) with hyperlipidemia (HLP). Detailed demographic information is presented in Table 1.

### Objective assessment of cardiac function

The NYHA classification categorized 28 cases (9.4%) as Class II, 129 cases (43.4%) as Class III, and 134 cases (45.1%) as Class IV. The mean LVEF value was 0.456 (standard error: 0.06), while the median level of NT-proBNP was 2925 pg/mL (IQR 1595–7784). For additional details, please see Table 1.

### Clinical, mortality, and rehospitalization outcomes

A total of 297 patients participated in this study. The longest follow-up period was 789 days, with a median follow-up duration of 333 days (IQR 169–509). And 107 (36.0%) instances of rehospitalization and 68 (22.9%) fatalities were recorded.

**Table 1:** Baseline data of patients with CHF

Characteristics	Total(*n*)	Mean/Median(Q1–Q3)/ Percentage
Gender (Male/Female) (%)	153/144	51.5%/48.5%
Age (Yeas old)	296	77(66–82)
Course of Disease (Years)	297	7(2-16.5)
Comorbidities		
Coronary Heart Disease	238	80.1%
Diabetes mellitus	109	36.7%
Hypertension	213	71.7%
Hyperlipidemia	182	61.3%
NYHA Classification	297	
I	6	2.0%
II	28	9.4%
III	129	43.4%
IV	134	45.1%
LVEF (%)	283	45.6% (10%)
NT-pro BNP (pg/mL)	192	7405(3791–30000)
Follow-up Time (Days)	294	333(169–509)
Prognosis	297	
Re-hospitalization	107	36.0%
End-Point	68	22.9%

### Correlation analysis

Spearman correlation analysis was employed to assess the relationship between cardiac function evaluation indices, renal function evaluation indices, and liver function evaluation indices, and the long-term prognosis of CHF patients (Table 2). The analysis reveals that higher Scr, BUN, UA, direct bilirubin (DBIL), NT-proBNP, NYHA classification, and cholesterol (CHOL) levels are associated with a worse long-term prognosis in CHF patients. Specifically, elevated levels of these indices are linked to a higher risk of mortality in patients with CHF. In contrast, platelet count (PLT), high-density lipoprotein cholesterol (HDL-C), and low-density lipoprotein cholesterol (LDL-C) demonstrate a negative correlation with the long-term prognosis of patients with CHF.

**Table 2:** Correlation analysis of cardiac function, renal function, and liver function with Long-Term prognosis in patients with CHF

	Spearman *r*	*P* value	*n*
Plt	-0.121	0.039*	293
LVEF	-0.093	0.119	280
Scr	0.181	0.001**	294
BUN	0.260	< 0.0001**	294
UA	0.184	0.002**	292
ALT	0.062	0.289	292
AST	0.076	0.194	292
TBIL	0.090	0.124	292
DBIL	0.117	0.046*	292
IBIL	0.074	0.206	292
BNP	0.190	0.008**	191
NYHA	0.128	0.036*	294
CHOL	0.001	− 0.199**	253
TG	0.368	-0.056	253
HDL-C	0.009	− 0.164**	253
LDL-C	0.004	− 0.179**	253
VLDL-C	0.427	0.051	253

*: *P* < 0.05, indicating significance, **:*P* < 0.01, indicating high significance

### Sensitivity analysis

**Fig. 1:**
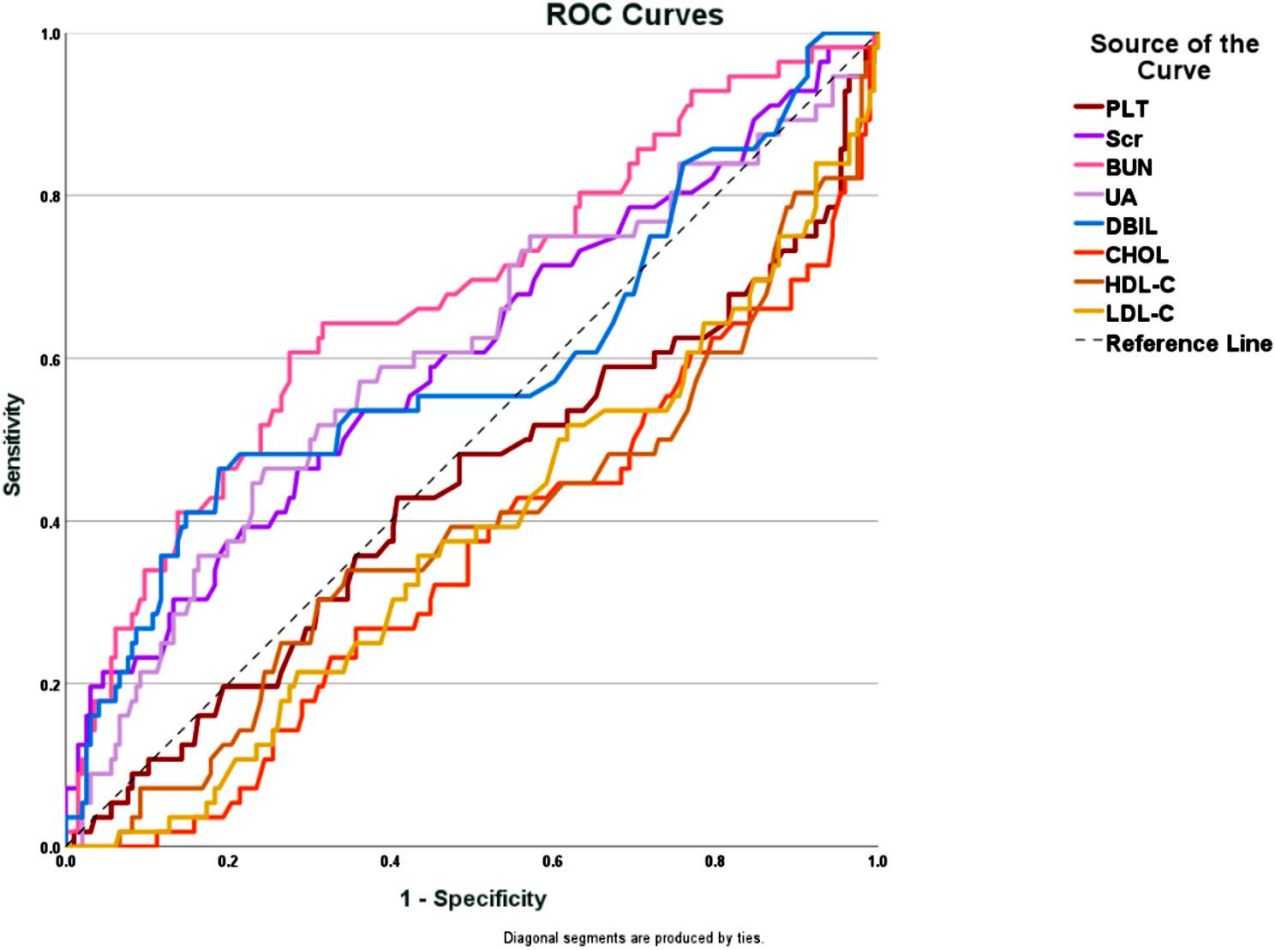
ROC curves for various biological indicators

The ROC curve associated with long-term prognosis was generated using SPSS 27.0 software. This analysis included PLT, Scr, BUN, UA, DBIL, CHOL, HDL-C, and LDL-C. The specific outcomes are illustrated in Fig. 1. The area under the curve (AUC) for serum creatinine was 0.602, with a P-value of 0.02. For BUN, the AUC was 0.674, with a P-value < 0.001; for UA, the AUC was also 0.602, with a P-value of 0.02. These results were statistically significant. In contrast, DBIL had a P-value greater than 0.05, indicating that it was not statistically significant. Additionally, the AUCs for PLT, CHOL, HDL-C, and LDL-C were all below 0.5, suggesting that their cut-off values have limited prognostic value (Table 3). By calculating 1 minus specificity and combining it with the sensitivity values from the software, we determined the optimal cut-off values: Scr at 101.5 µmol/L, BUN at 8.61 mmol/L, and UA at 462 µmol/L.

**Table 3:** Area under the ROC curve output

Test Result Variable(s)	Area	Std. Error^a^	Asymptotic Sig.^b^	Asymptotic 95% Confidence Interval	
				Lower Bound	Upper Bound
PLT	0.437	0.047	0.148	0.345	0.528
Scr	0.602	0.046	0.020*	0.512	0.691
BUN	0.674	0.043	0.000**	0.590	0.757
UA	0.602	0.046	0.020*	0.512	0.692
DBIL	0.592	0.048	0.051	0.498	0.686
CHOL	0.355	0.042	0.001**	0.271	0.438
HDL-C	0.390	0.046	0.012*	0.300	0.479
LDL-C	0.382	0.043	0.007**	0.297	0.466

*: *P* < 0.05, indicating significance, **:*P* < 0.01, indicating high significance

### Baseline data comparison of the two groups

**Fig. 2:**
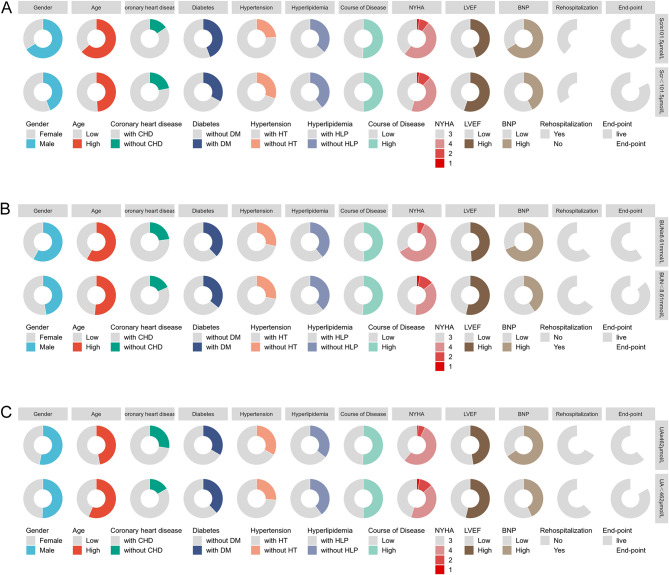
Comparison of baseline of CHF patients grouped by different Cut-off values of Scr, BUN, and UA

The baseline data, based on previously obtained cut-off values for Scr, BUN, and UA, were visualized using the ggplot2 package (version 3.4.4) in R Studio (version 4.2.1), as shown in Fig. 2. Appropriate statistical methods were employed for analysis using the stats (version 4.2.1) package according to the data characteristics, with the detailed results displayed in Tables 4, 5 and 6. The findings indicated significant differences in gender, age, NT-pro BNP levels, and mortality among patients grouped by different Scr groups. Significant variations were also observed in cardiac function classification, NT-pro BNP levels, and mortality among patients grouped by different BUN groups. Similarly, notable differences were observed in the prevalence of CHD, NT-pro BNP levels, and mortality among patients classified by different UA groups.

**Table 4:** Baseline comparison of CHF patients with different Scr groups

Characteristics (*n*)	Scr ≥ 101.5µmol/L(*n* = 97)	Scr<101.5µmol/L(*n* = 200)	*P* value
Gender, n (%)			< 0.001**
Female	33 (11.1%)	112 (37.7%)	
Male	64 (21.5%)	88 (29.6%)	
Age, median (Q1-Q3)	78 (70.75-83)	76 (65–80)	0.022*
Coronary heart disease, n (%)			0.186
with CHD	82 (27.6%)	156 (52.5%)	
without CHD	15 (5.1%)	44 (14.8%)	
Diabetes mellitus, n (%)			0.057
without DM	54 (18.2%)	134 (45.1%)	
with DM	43 (14.5%)	66 (22.2%)	
Hypertension, n (%)			0.223
with HT	74 (24.9%)	139 (46.8%)	
without HT	23 (7.7%)	61 (20.5%)	
Hyperlipidemia, n (%)			0.692
with HLP	61 (20.5%)	121 (40.7%)	
without HLP	36 (12.1%)	79 (26.6%)	
Course of Disease, median (Q1-Q3)	7 (2, 20)	6.5 (1.8325, 16)	0.618
NYHA, n (%)			0.627
IV	49 (16.5%)	85 (28.6%)	
III	38 (12.8%)	91 (30.6%)	
II	8 (2.7%)	20 (6.7%)	
I	2 (0.7%)	4 (1.3%)	
LVEF, median(Q1-Q3)	0.45 (0.39–0.52)	0.46 (0.4–0.54)	0.382
NT-pro BNP, median (Q1-Q3)	5326 (2291–14110)	2491 (1448–6272)	< 0.001**
Re-hospitalization, n (%)			0.431
Yes	38 (12.8%)	69 (23.2%)	
No	59 (19.9%)	131 (44.1%)	
End-point, n (%)			0.001**
live	64 (21.5%)	165 (55.6%)	
End-point	33 (11.1%)	35 (11.8%)	

*: *P* < 0.05, indicating significance, **:*P* < 0.01, indicating high significance

**Table 5:** Baseline comparison of CHF patients with different BUN groups

Characteristic (*n*)	BUN ≥ 8.61mmol/L(*n* = 105)	BUN<8.61mmol/L(*n* = 192)	*P* value
Gender, n (%)			0.078
Female	44 (14.8%)	101 (34%)	
Male	61 (20.5%)	91 (30.6%)	
Age, median(Q1-Q3)	78 (66–83)	77 (67-80.5)	0.269
Coronary heart disease, n (%)			0.339
with CHD	81 (27.3%)	157 (52.9%)	
without CHD	24 (8.1%)	35 (11.8%)	
Diabetes mellitus, n (%)			0.712
without DM	65 (21.9%)	123 (41.4%)	
with DM	40 (13.5%)	69 (23.2%)	
Hypertension, n (%)			0.935
with HT	75 (25.3%)	138 (46.5%)	
without HT	30 (10.1%)	54 (18.2%)	
Hyperlipidemia, n (%)			0.932
with HLP	64 (21.5%)	118 (39.7%)	
without HLP	41 (13.8%)	74 (24.9%)	
Course of Disease, median (Q1-Q3)	6 (3–18)	7 (1-15.25)	0.433
NYHA, n (%)			0.002**
IV	63 (21.2%)	71 (23.9%)	
III	35 (11.8%)	94 (31.6%)	
II	6 (2%)	22 (7.4%)	
I	1 (0.3%)	5 (1.7%)	
LVEF, median (Q1-Q3)	0.45 (0.3775–0.525)	0.46 (0.4–0.54)	0.195
NT-pro BNP, median (Q1-Q3)	6039.5 (2510–15404)	2413.5 (1472-5594.5)	< 0.001**
Re-hospitalization, n (%)			0.583
No	65 (21.9%)	125 (42.1%)	
Yes	40 (13.5%)	67 (22.6%)	
End-point, n (%)			< 0.001**
live	63 (21.2%)	166 (55.9%)	
End-point	42 (14.1%)	26 (8.8%)	

*: *P* < 0.05, indicating significance, **:*P* < 0.01, indicating high significance

**Table 6:** Baseline comparison of CHF patients with different UA groups

Characteristics (*n*)	UA ≥ 462µmol/L(*n* = 87)	UA<462µmol/L(*n* = 270)	*P* value
Gender, n (%)			0.707
Female	41 (13.8%)	104 (35%)	
Male	46 (15.5%)	106 (35.7%)	
Age, median(Q1-Q3)	76 (62-81.5)	78 (69–82)	0.171
Coronary heart disease, n (%)			0.032*
with CHD	63 (21.2%)	175 (58.9%)	
without CHD	24 (8.1%)	35 (11.8%)	
Diabetes mellitus, n (%)			0.438
without DM	58 (19.5%)	130 (43.8%)	
with DM	29 (9.8%)	80 (26.9%)	
Hypertension, n (%)			0.214
with HT	58 (19.5%)	155 (52.2%)	
without HT	29 (9.8%)	55 (18.5%)	
Hyperlipidemia, n (%)			0.482
with HLP	56 (18.9%)	126 (42.4%)	
without HLP	31 (10.4%)	84 (28.3%)	
Course of Disease, median(Q1-Q3)	7 (2.6665-16.5)	6.5 (2–16)	0.704
NYHA, n (%)			0.177
IV	47 (15.8%)	87 (29.3%)	
III	34 (11.4%)	95 (32%)	
II	5 (1.7%)	23 (7.7%)	
I	1 (0.3%)	5 (1.7%)	
LVEF, median (Q1-Q3)	0.45 (0.37-0.5225)	0.46 (0.4–0.54)	0.148
NT-pro BNP, median (Q1-Q3)	5145.5 (2008.2-13840)	2567 (1505.8-6466.2)	0.003**
Re-hospitalization, n (%)			0.534
No	58 (19.5%)	132 (44.4%)	
Yes	29 (9.8%)	78 (26.3%)	
End-point, n (%)			< 0.001**
live	54 (18.2%)	175 (58.9%)	
End-point	33 (11.1%)	35 (11.8%)	

*: *P* < 0.05, indicating significance, **:*P* < 0.01, indicating high significance

### Survival analysis

**Fig. 3:**
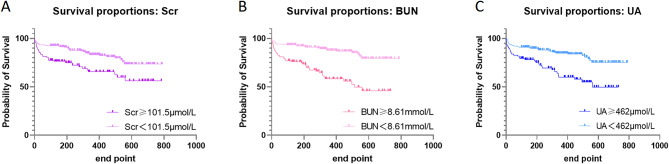
Kaplan-meier survival curves of CHF patients of different groups

Based on the cut-off values of Scr, BUN, and UA obtained from the ROC curve analysis, CHF patients were devided into two groups. Kaplan-Meier survival analysis was employed to illustrate the long-term prognosis of patients at different levels of Scr, BUN, and UA, as shown in Fig. 3. The figure demonstrates a clear separation between the two survival curves, highlighting significant differences in survival outcomes between the groups.

### Multivariate COX and logistic regression models

Proportional-hazards hypothesis testing and Cox regression analyses were conducted using the survival (version 3.3.1) and rms (version 6.3.0) packages in R studio (version 4.2.1). In the univariate analysis, samples meeting the predetermined P-value threshold were selected for the multivariate Cox regression model. Detailed results are presented in Table 7. The univariate Cox regression analysis identified several risk factors for poor prognosis: Scr ≥ 101.5 µmol/L (HR = 2.209, 95% CI 1.372–3.557, *P* = 0.001), BUN ≥ 8.61 mmol/L (HR = 3.709, 95% CI 2.270–6.061, *P* < 0.001), UA ≥ 462 µmol/L (HR = 2.625, 95% CI 1.631–4.228, *P* < 0.001), BNP levels (HR = 1.000, 95% CI 1.000–1.000, *P* < 0.001), male gender (HR = 1.764, 95% CI 1.067–2.915, *P* = 0.027), hyperlipidemia (HR = 0.567, 95% CI 0.351–0.916, *P* = 0.027), and re-hospitalization (HR = 0.480, 95% CI 0.280–0.826, *P* = 0.008). After adjustment, the factors included in the multivariate regression equation were: BUN ≥ 8.61 mmol/L (HR = 2.685, 95% CI 1.247–5.783, *P* = 0.012), coronary heart disease (HR = 3.291, 95% CI 1.139–9.511, *P* = 0.012), hyperlipidemia (HR = 0.468, 95% CI 0.271–0.811, *P* = 0.008), and re-hospitalization (HR = 0.316, 95% CI 0.131–0.765, *P* = 0.011).

**Table 7:** Results of proportional hazards regression models for risk factors in CHF patients

Characteristics	Total (*N*)	Univariate analysis			Multivariate analysis	
		Hazard ratio (95% CI)	*P* value		Hazard ratio (95% CI)	*P* value
Different Scr levels	294					
Scr<101.5µmmol/L	197	Reference			Reference	
Scr ≥ 101.5µmmol/L	97	2.209 (1.372–3.557)	0.001**		0.759 (0.349–1.651)	0.487
Different BUN levels	294					
BUN<8.61mmol/L	190	Reference			Reference	
BUN ≥ 8.61mmol/L	104	3.709 (2.270–6.061)	< 0.001**		2.685 (1.247–5.783)	0.012*
Different UA levels	294					
UA<462µmol/L	207	Reference			Reference	
UA ≥ 462µmol/L	87	2.625 (1.631–4.228)	< 0.001**		1.691 (0.796–3.596)	0.172
LVEF (%)	280	0.400 (0.039–4.145)	0.442			
NT-pro BNP (pg/mL)	191	1.000 (1.000–1.000)	< 0.001**		1.000 (1.000–1.000)	0.050
Gender	294					
Female	142	Reference			Reference	
Male	152	1.764 (1.067–2.915)	0.027*		1.647 (0.837–3.240)	0.148
Age (Years old)	294	1.014 (0.991–1.037)	0.233			
Coronary heart disease	294					
Without	235	Reference			Reference	
With	59	2.079 (0.994–4.350)	0.052		3.291 (1.139–9.511)	0.028*
Diabetes mellitus	294					
Without	107	Reference				
With	187	0.974 (0.592–1.601)	0.917			
Hypertension	294					
Without	211	Reference				
With	83	0.868 (0.519–1.453)	0.591			
Hyperlipidemia	294					
Without	181	Reference			Reference	
With	113	0.567 (0.351–0.916)	0.020*		0.468 (0.468)	0.027*
Course of Disease (Year)	294	1.011 (0.989–1.034)	0.328			
NYHA	294					
IV	133	Reference				
III	128	6461598.8579 (0.000 - Inf)	0.995			
II	27	7932290.7977 (0.000 - Inf)	0.995			
I	6	11554542.7867 (0.000 - Inf)	0.995			
Re-hospitalization	294					
No	187	Reference			Reference	
Yes	107	0.480 (0.280–0.826)	0.008*		0.316 (0.131–0.765)	0.011*

*: *P* < 0.05, indicating significance

### Subgroup analysis

**Fig. 4:**
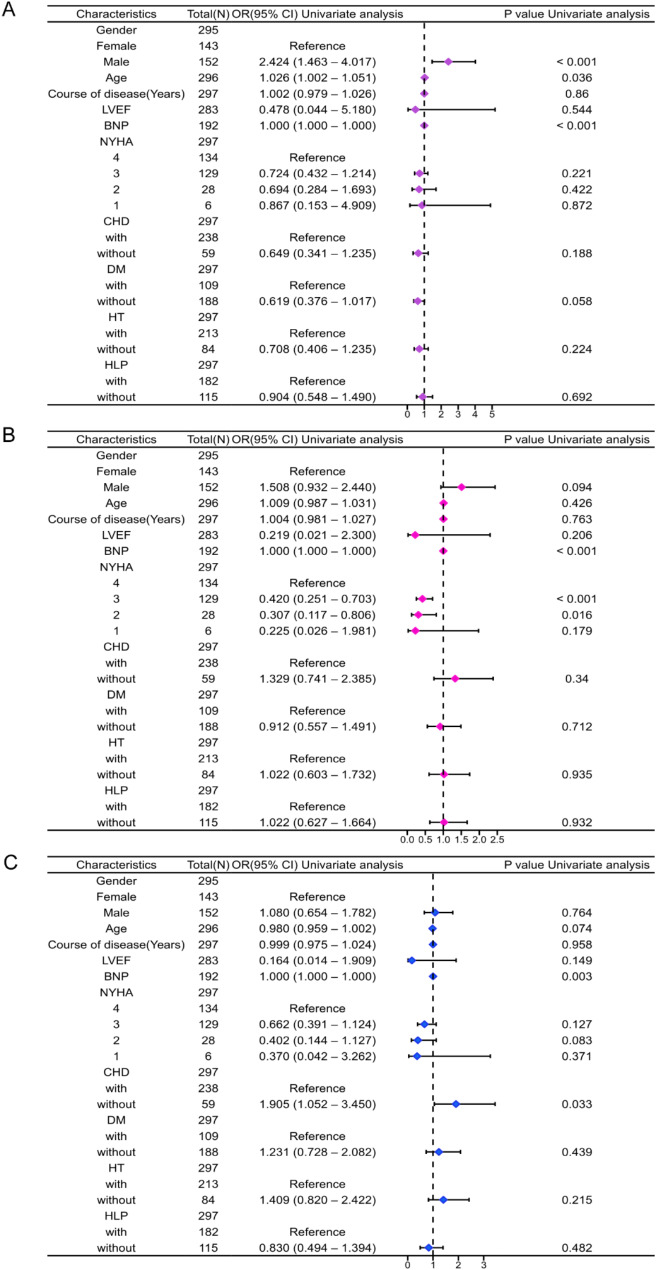
Subgroup forest analysis plots of different groups of Scr, BUN, and UA

Firstly, a univariate binary logistic regression analysis was conducted using rms (version 6.4.0) and ResourceSelection (version 0.3-5) packages in R studio (version 4.2.1) to assess how different levels of Scr, BUN, and UA affect the patients’ outcomes. The derived risk analysis values were used to create the subgroup forest plot, and the results were visualized through ggplot2 (version 3.4.4), as shown in Fig. 4.

Among different Scr cut-off groups, male patients had a hazard ratio (HR) of 2.424 compared to female patients (95% CI: 1.463–4.017, *P* < 0.001), while the HR related to age was 1.026 (95% CI: 1.002–1.051, *P* = 0.036). These findings indicate that male patients have a HR of 2.424 for adverse outcomes compared to female patients, and the prognosis worsens with advancing age. The HR for BNP was 1.000 (*P* < 0.001), while the HR for the transition from NYHA class III to class IV was 0.42 (95% CI: 0.251–0.704, *P* < 0.001). Additionally, the HR for NYHA class II to NYHA class III was 0.307 (95% CI: 0.117–0.806, *P* = 0.016). These data suggest that worsening cardiac function class is associated with poorer patient outcomes. Patients without CHD had a HR of 1.905 for adverse outcomes compared to those with CHD (95% CI: 1.052–3.405, *P* = 0.033). All these results were statistically significant.

The subgroup analyses show that BNP levels significantly affect patient prognosis, and factors like gender, age, and cardiac function classification also contribute to prognosis, consistent with previous studies.

### Medication bias correction

The study employs a real-world model for its analysis. Patients received treatment according to the 2014 Chinese Guidelines for Chronic Heart Failure, which may involve thiazide diuretics that may impair UA levels. Consequently, in this study, we performed a correlation analysis and adjusted COX regression analysis to eliminate medication-related bias, which showed that thiazides had no significant effect on UA levels in CHF patients. Detailed analyses are provided in the Supplementary Material.

## Discussion

CHF is prevalent worldwide, greatly affecting patients’ quality of life and bringing a significant economic burden on society. Understanding the factors influencing long-term prognosis could help clinicians implement early interventions. Emerging biomarkers have shown great potential in predicting the prognosis of CHF. For instance, a study indicated that LGI, calculated by multiplying blood glucose levels by white blood cell count, was significantly associated with in-hospital mortality in critical care unit (CCU) patients, particularly when it exceeded 3.72 [17]. This study’s strength lies in its systematic analysis of various biomarkers and their interrelationships, offering a prognostic evaluation method which can be widely applied in clinical practice, particularly in patients’ primary care. Our findings show that Scr, BUN, and UA levels are critical indicators impacting patients’ outcomes. Although previous research has shown that renal dysfunction, indicated by high Scr, is linked to increased mortality in CHF patients [22], this study not only supports existing findings but also explores the roles of BUN and UA levels as supplementary prognostic factors, aiming to broaden the understanding of these markers’ potential utility in managing CHF and ultimately assist clinicians in developing personalized treatment plans.

This study incorporated various biochemical markers, including PLT, AST, ALT, DBIL, TBIL, CHOL, TG, LDL-C, HDL-C, VLDL-C, Scr, BUN, and UA, to investigate their relationship with CHF prognosis. Subsequently, ROC curve analysis of the selected biochemical markers revealed that only Scr, BUN, and UA had significant predictive value for the long-term prognosis of CHF. Scr, a byproduct of muscle metabolism which primarily excreted by the kidneys, usually maintains a stable concentration under normal physiological conditions, indicating an individual’s muscle mass and renal function [23]. Scr level fluctuations in CHF patients are believed to reflect changes in cardiac workload and renal stress response [24]. Findings from a TOPCAT study demonstrated a significant link between elevated Scr levels and increased all-cause mortality in heart failure patients [25], emphasizing the critical importance of monitoring Scr level in clinical evaluation. The Scr threshold for predicting the long-term prognosis of CHF is 101.5 µmol/L in this study, lower than the standard Scr threshold of 133 µmol/L for diagnosing renal dysfunction [26], sugguesting that clinicians should be particularly cautious when Scr reaches 101.5 µmol/L in CHF treatment, as early intervention could significantly improve patient outcomes. BUN, a key byproduct of protein metabolism primarily eliminated by the kidneys, serves as an indicator of renal function. Elevated BUN level usually indicates impaired renal function. Research has shown that there is a strong correlation between BUN level and cardiac output, as well as hydration status, suggesting BUN is associated with overall prognosis of CHF patients. This investigation involved a comprehensive review and meta-analysis of 19 cohort studies including 56,003 CHF patients, revealing that elevated BUN level is an independent prognostic factor for all-cause mortality in these patients [27]. And it also noted that all-cause mortality risk increases significantly when BUN exceeds 25 mg/dL (8.925 mmol/L), which aligns with the BUN threshold identified in our study. Furthermore, BUN exhibits an inverse relationship with LVEF [23], reduced LVEF is often associated with elevated BUN, indicating that cardiac dysfunction may lead to renal under-perfusion, which in turn increases BUN. UA serves as the terminal metabolite of purine catabolism, it circulates freely in the body and acts as an antioxidant, helping in neutralizing free radicals and reducing cellular damage caused by oxidative stress. Additionally, UA contributes to cardiovascular health through the stimulation of endothelial cell proliferation and migration, also involves in vascular repair and regeneration processes [28]. Hyperuricemia predicts cardiovascular disease and is significantly associated with heart failure and mortality [29, 30]. Elevated UA levels are posited to exacerbate heart failure via mechanisms that enhance oxidative stress, promote inflammation, and impair myocardial cell function [31]. Furthermore, increased UA may compromise the efficacy of diuretic treatments, necessitating higher dosages of diuretics for hospitalized heart failure patients [32]. Consequently, monitoring UA could help to assess the status and predict the prognosis of CHF patients.

The study shows that among the numerous factors influencing the long-term prognosis of CHF, renal function exhibits the predominant influence. Recent studies show that renal function is intricately connected with the severity of CHF, as well as patients’ therapeutic response and long-term outcomes. This relationship between the cardiovascular and renal systems is known as cardiorenal syndrome (CRS) [33]. The pathophysiology of CRS is complex and involves various physiological and pathological mechanisms. Renal impairment can result in fluid retention and electrolyte disturbances, thereby exacerbating the workload on the heart and leading to progressive cardiac dysfunction. Concurrently, heart failure often leads to circulatory hypoperfusion, alterations in endogenous hormone levels, and adverse drug reactions, which in turn impair renal function and result in a harmful feedback loop between heart and kidney [34, 35]. This bidirectional relationship underscores the urgent need for multidisciplinary collaboration. However, changes in renal function are often overlooked in clinical practice. Many clinicians may reduce or stop renin-angiotensin-aldosterone system (RAAS) inhibitors too soon when noticing the renal function decline, worsening the prognosis of CHF patients [36]. Therefore, the evaluation and management of renal function should be included in the core treatment for CHF patients to enhance overall therapeutic efficacy. ARNI drugs should be given priority to improve cardio-renal hemodynamics, dynamic monitoring of estimated glomerular filtration rate (eGFR) [37, 38], and adjusting diuretics doses. Abnormal UA level reflects the activation of oxidative stress-inflammation axis [39]. Allopurinol can target the xanthine oxidase pathway, which may reduce UA and improve myocardial energy metabolism [40, 41]. Additionally, new medications and therapeutic approaches like sodium-glucose cotransporter 2 (SGLT2) inhibitors [42, 43] improve both renal and heart function in CHF patients, thus further enhancing their long-term prognosis.

Høfsten DE et al. pointed out that the annual increase in UA for CHF patients using thiazides was 0.12 mg/dL higher than for those not using thiazides over a 5-year follow-up [44]. Another study revealed that in CHF patients, long-term use of thiazide (25–50 mg/day) leads to a dose-dependent increase in UA levels, averaging 0.8–1.5 mg/dL [45]. However, in this study, we included thiazides application into correlation analysis, multivariate Cox regression, subgroup analysis and survival analysis, results show that whether patients take thiazides or not does not affect their UA level and long-term prognosis. This study focuses on a real-world population and employs a non-intervention-based treatment regimen. CHF Patients often have comorbidities, such as hyperuricemia, leading to the use of allopurinol and other medications to lower UA levels, which can reduce the effectiveness of thiazides in raising UA. Additionally, this study utilizes real-world data, and the determined UA cut-off values accurately reflect UA levels in patients with CHF.

However, this study has certain limitations. The limited study population and the single-center design might restrict the application of our findings to a wider population. And the study’s retrospective nature may introduce bias because it relies on existing clinical records, preventing real-time observation or intervention. Furthermore, the lack of multicenter validation reduces the external validity of our results. Finally, although we have identified key risk factors associated with adverse outcomes, the absence of experimental validation hinders the establishment of a causal relationship. Future studies should focus on large-scale multicenter trials that include long-term follow-ups to evaluate the long-term effects of these biomarkers on clinical outcomes in CHF [46]. Furthermore, addressing these limitations could enhance our understanding of the prognostic value of biomarkers such as Scr, BUN, and UA in managing CHF.

## Conclusion

In summary, our study underscores the pivotal relationship between biochemical indicators, clinical characteristics, and prognosis in patients with CHF. By identifying key risk factors such as Scr, BUN and UA cut-off values, we provide valuable insights into clinical decision-making. These results highlight the need for personalized management strategies to improve outcomes in those high-risk population. Additionally, this article synthesizes and reevaluates the commonly utilized biomarkers to investigate their predictive value for the long-term prognosis of CHF, holding significant implications for guiding CHF management in primary healthcare. Our future research endeavors should focus on expanding study population and conducting prospective studies to confirm these correlations, thereby facilitating the development of more efficacious therapeutic approaches for CHF patients and enhancing the overall standard of care.

## Electronic supplementary material

Below is the link to the electronic supplementary material.

Supplementary Material 1

## Data Availability

The data will be available on reasonable request.

## Abbreviations

CHF: Chronic Heart Failure
NT-proBNP: Serum N-Terminal Pro-Brain Natriuretic Peptide
NYHA: New York Heart Association
LVEF: Left Ventricular Ejection Fraction
HA: Hyaluronic Acid
LGI: Lactate Glucose Index
Scr: Serum creatinine
BUN: Blood Urea Nitrogen
UA: Uric Acid
ROC: Receiver Operating Characteristic
AUC: Area Under the Curve
HR: Hazard Ratio
CHD: Coronary Heart Disease
HT: Hypertension
DM: Diabetes Mellitus
HLP: Hyperlipidemia
CRS: Cardiorenal Syndrome
