# Supplementary material for: The role of cut-off values for creatinine, blood urea nitrogen, and uric acid in prognostic assessment of chronic heart failure: a retrospective cohort study
Source: BMC Cardiovasc Disord. 2025 Mar 22;25:209. doi: 10.1186/s12872-025-04675-y (PMC11929248; doi:10.1186/s12872-025-04675-y)

**Supplementary Materials**

1. **Descriptive analysis and correlation analysis of thiazides application**

Among the 297 patients, 118 (39.7%) used thiazides to lower cardiac preload. The correlation analysis indicated a coefficient of -0.028 for the relationship between thiazide use and serum uric acid level, with a P value of 0.638. Additionally, the correlation coefficient between the use of thiazides and death was -0.03, with a P value of 0.606. Consequently, we concluded that thiazide use did not significantly affect the risk of death in this study, and thus, we excluded it from the ROC curve analysis. The results of descriptive analysis and correlation analysis are presented in the following two tables.

Table1-Baseline Data of Patients with CHF

| Characteristics | Total(n) | Mean/Median(Q1–Q3)/Percentage |
| --- | --- | --- |
| Application of thiazides | 118/297 | 39.7% |

Table2-Correlation Analysis of Application of Thiazides with Serum Uric Acid level and Long-Term Prognosis in Patients with CHF

| **Correlations** | | | | | |
| --- | --- | --- | --- | --- | --- |
|  |  |  | use thiazides | End-point(Death) | serum uric acid |
| Spearman's rho | use thiazides | Correlation Coefficient | 1.000 | -0.030 | -0.028 |
|  |  | Sig. (2-tailed) |  | 0.606 | 0.638 |
|  |  | N | 297 | 294 | 295 |

1. **Multivariate COX regression**

Thiazides application was included as a mandatory co-variate in the multivariate Cox proportional hazards model, and the COX regression model was recalculated and presented in Table 3. The univariate Cox regression analysis demonstrated the Thiazides application as a risk factors for poor prognosis: HR 0.938, 95% CI 0.575 - 1.530 (*P = 0.798*), and has not been included in the multivariate analysis model. We can see that the inclusion of thiazide in the COX regression model did not affect the statistical results of other factors in the original manuscript.

Table3-Results of Proportional Hazards Regression Models for Risk Factors in CHF Patients

| Characteristics | Total(N) | Univariate analysis | |  | Multivariate analysis | |
| --- | --- | --- | --- | --- | --- | --- |
|  |  | Hazard ratio (95% CI) | P value |  | Hazard ratio (95% CI) | P value |
| Different Scr levels | 294 |  |  |  |  |  |
| Scr＜101.5μmmol/L | 197 | Reference |  |  | Reference |  |
| Scr≥101.5μmmol/L | 97 | 2.209 (1.372 - 3.557) | **0.001** |  | 0.759 (0.349 - 1.651) | 0.487 |
| Different BUN levels | 294 |  |  |  |  |  |
| BUN＜8.61mmol/L | 190 | Reference |  |  | Reference |  |
| BUN≥8.61mmol/L | 104 | 3.709 (2.270 - 6.061) | **< 0.001** |  | 2.685 (1.247 - 5.783) | **0.012** |
| Different UA levels | 294 |  |  |  |  |  |
| UA＜462μmol/L | 207 | Reference |  |  | Reference |  |
| UA≥462μmol/L | 87 | 2.625 (1.631 - 4.228) | **< 0.001** |  | 1.691 (0.796 - 3.596) | 0.172 |
| LVEF | 280 | 0.400 (0.039 - 4.145) | 0.442 |  |  |  |
| BNP | 191 | 1.000 (1.000 - 1.000) | **< 0.001** |  | 1.000 (1.000 - 1.000) | 0.050 |
| Gender | 294 |  |  |  |  |  |
| female | 142 | Reference |  |  | Reference |  |
| male | 152 | 1.764 (1.067 - 2.915) | **0.027** |  | 1.647 (0.837 - 3.240) | 0.148 |
| Age | 294 | 1.014 (0.991 - 1.037) | 0.233 |  |  |  |
| Coronary heart disease | 294 |  |  |  |  |  |
| without | 59 | Reference |  |  | Reference |  |
| with | 235 | 2.079 (0.994 - 4.350) | 0.052 |  | 3.291 (1.139 - 9.511) | **0.028** |
| Diabetes | 294 |  |  |  |  |  |
| without | 187 | Reference |  |  |  |  |
| with | 107 | 0.974 (0.592 - 1.601) | 0.917 |  |  |  |
| Hypertension | 294 |  |  |  |  |  |
| without | 83 | Reference |  |  |  |  |
| with | 211 | 0.868 (0.519 - 1.453) | 0.591 |  |  |  |
| Hyperlipidemia | 294 |  |  |  |  |  |
| without | 113 | Reference |  |  | Reference |  |
| with | 181 | 0.567 (0.351 - 0.916) | **0.020** |  | 0.468 (0.239 - 0.917) | **0.027** |
| (Year) | 294 | 1.011 (0.989 - 1.034) | 0.328 |  |  |  |
| NYHA | 294 |  |  |  |  |  |
| Ⅰ | 6 | Reference |  |  |  |  |
| Ⅱ | 27 | 6461598.8579 (0.000 - Inf) | 0.995 |  |  |  |
| Ⅲ | 128 | 7932290.7977 (0.000 - Inf) | 0.995 |  |  |  |
| Ⅳ | 133 | 11554542.7867 (0.000 - Inf) | 0.995 |  |  |  |
| Hospital readmission | 294 |  |  |  |  |  |
| No | 187 | Reference |  |  | Reference |  |
| Yes | 107 | 0.480 (0.280 - 0.826) | **0.008** |  | 0.316 (0.131 - 0.765) | **0.011** |
| Thiazides | 294 |  |  |  |  |  |
| Not taken | 179 | Reference |  |  |  |  |
| Taken | 115 | 0.938 (0.575 - 1.530) | 0.798 |  |  |  |

1. **Subgroup analysis**

In the subgroup without thiazides (n=197), a UA level of ≥462 mmol/L was associated with a 2.41-fold increased risk of end-point event in CHF patients (95% CI 1.311-4.430, *P=0.005*) (Table 4), while in the treatment group (n=115), this risk was 2.97 times higher (95% CI 1.372-6.436, *P=0.006*) (Table 5), The interaction term yielded a p-value greater than 0.05, demonstrating that the hazard ratio for UA does not significantly differ between the treated and untreated groups. This consistent dose-response relationship indicates that UA has a pathological effect independent of thiazide treatment.

Table 4-Regression model for risk factors in CHF patients without thiazides application

| Characteristics | Total(N) | Univariate analysis | |  | Multivariate analysis | |
| --- | --- | --- | --- | --- | --- | --- |
|  |  | Hazard ratio (95% CI) | P value |  | Hazard ratio (95% CI) | P value |
| Different Scr levels | 179 |  |  |  |  |  |
| Scr＜101.5μmmol/L | 119 | Reference |  |  | Reference |  |
| Scr≥101.5μmmol/L | 60 | 1.734 (0.940 - 3.199) | 0.078 |  | 0.727 (0.300 - 1.759) | 0.480 |
| Different BUN levels | 179 |  |  |  |  |  |
| BUN＜8.61mmol/L | 115 | Reference |  |  | Reference |  |
| BUN≥8.61mmol/L | 64 | 2.869 (1.554 - 5.297) | **< 0.001** |  | 1.882 (0.680 - 5.212) | 0.223 |
| Different UA levels | 179 |  |  |  |  |  |
| UA＜462μmol/L | 128 | Reference |  |  | Reference |  |
| UA≥462μmol/L | 51 | 2.410 (1.311 - 4.430) | **0.005** |  | 1.727 (0.717 - 4.162) | 0.223 |
| LVEF | 174 | 0.318 (0.017 - 5.999) | 0.444 |  |  |  |
| BNP | 119 | 1.000 (1.000 - 1.000) | **< 0.001** |  | 1.000 (1.000 - 1.000) | **0.005** |
| Gender | 179 |  |  |  |  |  |
| female | 97 | Reference |  |  |  |  |
| male | 82 | 1.013 (0.549 - 1.868) | 0.968 |  |  |  |
| Age | 179 | 1.021 (0.992 - 1.052) | 0.162 |  |  |  |
| Coronary heart disease | 179 |  |  |  |  |  |
| without | 40 | Reference |  |  | Reference |  |
| with | 139 | 3.306 (1.178 - 9.278) | **0.023** |  | 3.427 (1.016 - 11.558) | **0.047** |
| Diabetes | 179 |  |  |  |  |  |
| without | 109 | Reference |  |  |  |  |
| with | 70 | 1.178 (0.636 - 2.184) | 0.602 |  |  |  |
| Hypertension | 179 |  |  |  |  |  |
| without | 56 | Reference |  |  |  |  |
| with | 123 | 0.829 (0.441 - 1.559) | 0.560 |  |  |  |
| Hyperlipidemia | 179 |  |  |  |  |  |
| without | 70 | Reference |  |  |  |  |
| with | 109 | 0.672 (0.364 - 1.241) | 0.204 |  |  |  |
| (Year) | 179 | 1.016 (0.988 - 1.044) | 0.276 |  |  |  |
| NYHA | 179 |  |  |  |  |  |
| Ⅰ | 4 | Reference |  |  |  |  |
| Ⅱ | 18 | 7978335.7894 (0.000 - Inf) | 0.996 |  |  |  |
| Ⅲ | 82 | 8186997.6239 (0.000 - Inf) | 0.996 |  |  |  |
| Ⅳ | 75 | 10994455.6100 (0.000 - Inf) | 0.996 |  |  |  |
| Hospital readmission | 179 |  |  |  |  |  |
| No | 115 | Reference |  |  | Reference |  |
| Yes | 64 | 0.462 (0.231 - 0.922) | **0.029** |  | 0.407 (0.150 - 1.100) | 0.076 |

Table 5-Regression model for risk factors in CHF patients with thiazides application

| Characteristics | Total(N) | Univariate analysis | |  | Multivariate analysis | |
| --- | --- | --- | --- | --- | --- | --- |
|  |  | Hazard ratio (95% CI) | P value |  | Hazard ratio (95% CI) | P value |
| Different Scr levels | 115 |  |  |  |  |  |
| Scr＜101.5μmmol/L | 78 | Reference |  |  | Reference |  |
| Scr≥101.5μmmol/L | 37 | 3.314 (1.520 - 7.226) | **0.003** |  | 1.315 (0.544 - 3.176) | 0.543 |
| Different BUN levels | 115 |  |  |  |  |  |
| BUN＜8.61mmol/L | 75 | Reference |  |  | Reference |  |
| BUN≥8.61mmol/L | 40 | 5.879 (2.537 - 13.620) | **< 0.001** |  | 4.857 (1.832 - 12.880) | **0.001** |
| Different UA levels | 115 |  |  |  |  |  |
| UA＜462μmol/L | 79 | Reference |  |  | Reference |  |
| UA≥462μmol/L | 36 | 2.971 (1.372 - 6.436) | **0.006** |  | 1.090 (0.457 - 2.601) | 0.846 |
| LVEF | 106 | 0.520 (0.011 - 24.329) | 0.739 |  |  |  |
| BNP | 72 | 1.000 (1.000 - 1.000) | 0.880 |  |  |  |
| Gender | 115 |  |  |  |  |  |
| female | 60 | Reference |  |  | Reference |  |
| male | 55 | 4.985 (1.878 - 13.228) | **0.001** |  | 4.398 (1.601 - 12.076) | **0.004** |
| Age | 115 | 1.004 (0.969 - 1.040) | 0.822 |  |  |  |
| Coronary heart disease | 115 |  |  |  |  |  |
| without | 19 | Reference |  |  |  |  |
| with | 96 | 1.018 (0.350 - 2.957) | 0.974 |  |  |  |
| Diabetes | 115 |  |  |  |  |  |
| without | 78 | Reference |  |  |  |  |
| with | 37 | 0.683 (0.287 - 1.628) | 0.390 |  |  |  |
| Hypertension | 115 |  |  |  |  |  |
| without | 27 | Reference |  |  |  |  |
| with | 88 | 1.003 (0.401 - 2.508) | 0.995 |  |  |  |
| Hyperlipidemia | 115 |  |  |  |  |  |
| without | 43 | Reference |  |  | Reference |  |
| with | 72 | 0.441 (0.202 - 0.961) | **0.039** |  | 0.484 (0.220 - 1.066) | 0.072 |
| (Year) | 115 | 1.003 (0.966 - 1.042) | 0.874 |  |  |  |
| NYHA | 115 |  |  |  |  |  |
| Ⅰ | 2 | Reference |  |  |  |  |
| Ⅱ | 9 | 3681773.7005 (0.000 - Inf) | 0.997 |  |  |  |
| Ⅲ | 46 | 7408440.4292 (0.000 - Inf) | 0.997 |  |  |  |
| Ⅳ | 58 | 12702212.4684 (0.000 - Inf) | 0.997 |  |  |  |
| Hospital readmission | 115 |  |  |  |  |  |
| No | 72 | Reference |  |  |  |  |
| Yes | 43 | 0.524 (0.219 - 1.252) | 0.146 |  |  |  |

1. **Survival analysis**

The analysis of survival curves for thiazide use demonstrated no significant differences, with a P value of 0.74 indicating a lack of statistical significance.


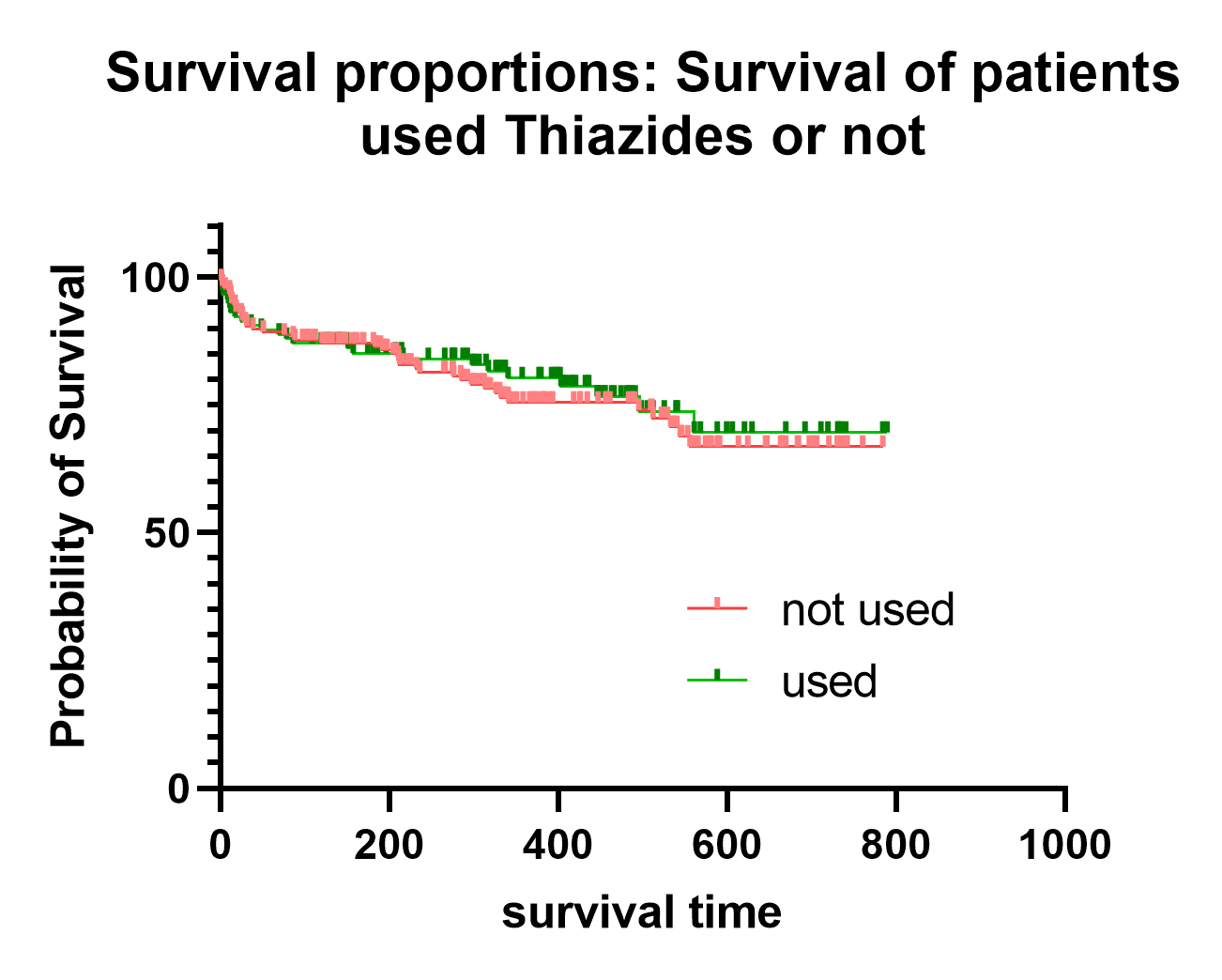

Supplement: Supplementary file 1 — Supplementary Material 1 [file 12872_2025_4675_MOESM1_ESM.docx]
